# Gender differences in the effects of economic inequality on mental conditions: a multivariate meta-analysis of Gini index and relative income position in high-income OECD countries

**DOI:** 10.3389/fpsyt.2026.1835495

**Published:** 2026-09-03

**Authors:** Olivia Kalinowski, Wulf Rössler

**Affiliations:** 1 Department of Psychiatry and Neurosciences, Psychiatric University Clinic of Charité at St. Hedwig Hospital, Berlin, Germany; 2 Nuffield Department of Primary Care Health Sciences, University of Oxford, Oxford, United Kingdom; 3 Department of Psychiatry and Psychotherapy, Psychiatric University Hospital Zurich, University of Zurich, Zurich, Switzerland

**Keywords:** depression, difference, gender, Gini coefficient, inequality, poverty, sex

## Abstract

**Background:**

Economic inequalities are established determinants of mental conditions, yet the role of gender remains insufficiently examined. In this article, we suggest that women experience a “double risk” in contexts of economic inequality: higher exposure to economic disadvantage and greater vulnerability to its mental health effects.

**Methods:**

We conducted a two-step synthesis. First, we analyzed sex differences in relative poverty across high-income OECD countries. Mean differences were assessed using Welch t-tests, with sensitivity analyses across COVID-19 periods. Second, we performed a systematic review and multivariate random-effects meta-analysis following PRISMA guidelines. We included 21 studies reporting gender-disaggregated associations between income rank and income inequality and ICD-/DSM-based mental conditions. Effect sizes were transformed to log odds ratios and analyzed using multilevel models accounting for within-study dependencies. Gender was examined as a moderator.

**Results:**

Women exhibited higher relative poverty rates than men (20.5% vs. 18.3%; mean difference: 2.2 percentage points; p < 0.001). The meta-analysis (46 effect sizes) showed a significant association between lower income and poorer mental health (log(OR)=0.55, 95% CI 0.26–0.85), with substantial heterogeneity. Gender did not moderate effects across all outcomes. However, in depression-specific analyses (k=17), gender significantly moderated the association (p=0.016), with stronger effects in women. Income inequality (Gini index) showed no overall association with mental health, but gender still emerged as a significant moderator, with effects concentrated among women in adjusted models.

**Conclusions:**

Gender shapes both exposure to economic disadvantage and vulnerability to its mental health consequences. While income-related mental health inequalities affect both sexes, women appear disproportionately impacted, particularly regarding depression. These findings highlight the importance of integrating gender-sensitive approaches into research and policy addressing socioeconomic determinants of mental health.

**Systematic review registration:**

https://www.crd.york.ac.uk/PROSPERO/view/CRD420251123692, identifier CRD420251123692.

## 1 Introduction

Economic inequalities are increasingly recognized as key determinants of mental conditions, yet the role of gender remains underexplored (1–6). Gender differences in the prevalence of mental conditions among socioeconomically disadvantaged populations are frequently reported, but rarely examined as a central analytic dimension (6). Ribeiro et al. (1), e.g., reported a pooled Cohen’s d effect size of 0.12 (95% CI: 0.05–0.20) for the association between income inequality and depression in their meta-analysis, yet gender was not examined as a potential moderator, despite the availability of gender-disaggregated data in much of the existing literature (6). Therefore, this present analysis aims to explore the role of gender within the relationship between economic inequalities and mental conditions.

Based on the available literature, we suggest in this article that gender is related to worse mental health outcomes in two distinct ways and hypothesize that women experience a double risk for mental conditions in contexts of economic inequality: first, through higher exposure to economic disadvantage, and second, through stronger mental health effects associated with both income inequality and relative economic position. Furthermore, we argue that gendered economic disparities may contribute to observed gender differences in mental disorder prevalence, particularly depression, which remains a central and unresolved debate in psychiatric epidemiology.

### 1.1 Economic inequality

Numerous heterogeneous concepts exist to conceptualize and measure economic inequality (6). In this analysis, we focus on two major interrelated concepts. First, we utilize the *Gini index* (also known as *Gini coefficient)*, which is the most common and standardized measure for *income inequality*. The Gini index is a summary measure of income (or wealth) inequality that ranges from 0 to 1, where 0 represents perfect equality, and 1 represents maximal inequality.

Second, we operationalize economic disadvantage at the individual level using income-based quartiles and quintiles, comparing individuals in the lowest versus highest income groups (Q1 vs Q4/Q5). This approach captures relative economic position within populations rather than *absolute poverty*. While conceptually related to *relative poverty*, commonly defined as earning 50–60% of a society’s median income, income quantile comparisons allow for a more continuous assessment of gradients in mental health across the income distribution. Finally, we restrict our analysis to high-income OECD countries to minimize conceptual overlap between *absolute* and *relative poverty*, as in lower-income settings, material deprivation often co-occurs with relative disadvantage, potentially obscuring effects on mental conditions.

### 1.2 Economic inequality and mental conditions

Elevated Gini index as well as low relative economic status have both been linked to heightened stress, limited access to social resources, and an increased burden of mental illness, most consistently depression, but also anxiety and psychotic disorders, across all age groups, including children, adolescents, adults, and older adults (1, 6).

Several theoretical frameworks have been proposed to explain these associations. The Status Anxiety Hypothesis highlights the detrimental effects of perceived social inferiority and chronic comparison stress (7, 8). The Social Causation Thesis argues that economic hardship directly contributes to mental illness via stress and reduced coping resources (9–11), whereas the Social Drift (Selection) Hypothesis conversely posits that mental illness can itself precipitate downward social mobility, leading to poverty (9, 12, 13). Other frameworks, such as the Social Mobility Hypothesis, underscore that already the expectations of upward or downward mobility bear risk or resilience effects (14–17). Empirical findings suggest that these mechanisms may interact and that causality may be bidirectional and context-dependent, varying across developmental stages and socioeconomic systems (6, 18, 19). Existing data suggest, e.g., bidirectional causal pathways between subjective financial status and disorders such as depression and alcohol misuse (19). Yet the predominance of cross-sectional designs in this field prevents conclusions about causality (6).

Previous research highlighted that economic inequality affects both adults and children/adolescents to a similar degree, even though mechanisms inherently differ across age groups (6). While the social drift hypothesis (mental illness leading to lower economic status) cannot explain poverty among children, who experience deprivation through their family environment, social causation (lower economic status leading to mental illness) may contribute to deteriorating mental conditions of children. Longitudinal studies in youth populations indicate that persistent poverty predicts behavioral and emotional problems, particularly when parental education is low (20, 21).

### 1.3 The role of gender

“Sex” usually describes biological characteristics (e.g., male, female), whereas “gender” refers to socially constructed identities, roles, and expressions. In this article, most included studies reported biological sex (male/female). We retain the terminology used in the original studies when describing their results, while interpreting these findings within the broader framework of gender-related differences. Gender seems to be related to economic inequality and mental illness in two distinct ways. First, across the literature, we observe gender differences in exposure to relative poverty. Wherever women are exposed more often to relative poverty, this may partially account for the higher prevalence of mental illness observed among women, given the fact that relative poverty is an established risk factor for mental conditions (see 1.2).

However, even when comparing women and men within the same income categories, higher prevalences of mental disorders are often observed among women and girls. For instance, a large Canadian cohort study of more than 190.000 adolescents showed that, among those exposed to low family income, the risk of non-affective psychotic disorders was higher for girls than for boys (adjusted hazard ratio [aHR] girls = 1.97; boys = 1.70). This pattern contrasts with most epidemiological evidence, which generally reports higher incidence rates and earlier onset of non-affective psychotic disorders among males than females, particularly in adolescence and early adulthood (22).

We suggest two main explanations to approach this discrepancy in effect sizes:

1. From a biomedical perspective, biological sex and gender-related factors may modify vulnerability through biological and physiological mechanisms, including differences in hormonal regulation, stress reactivity, neurodevelopment, and immune–endocrine interactions, which may render girls and women more sensitive to chronic psychosocial stressors associated with economic disadvantage. Evidence for gender-specific stress responses, including heightened hypothalamic–pituitary–adrenal (HPA) axis reactivity and differential patterns of affective regulation, supports this view (23, 24).
2. From a socio-economic and cultural perspective, we can observe that gender correlates with other variables, which then cause the effect, such as e.g. domestic violence (25).

These perspectives are not mutually exclusive and likely interact across the life course, highlighting the need for integrative explanatory models that account for both gender-differences in exposure risk to relative poverty, as well as gender differences in vulnerability to effects of economic inequality.

Gender differences are particularly pronounced for depressive disorders, where women consistently show higher prevalence across most populations and age groups. Large epidemiological surveys and global burden estimates indicate that women are approximately 1.5–2 times more likely than men to experience depression over the life course, a pattern observed across high-income settings and summarized by the World Health Organization. While part of this disparity has been attributed to differential help-seeking and diagnostic practices, converging evidence suggests that structural and psychosocial factors play a substantial role, including gendered exposure to economic insecurity, caregiving burden, interpersonal violence, and chronic stress, reinforcing the relevance of examining gender as a moderator in the inequality–depression relationship.

### 1.4 Research questions and aims

The aim of this article is to clarify the role of gender in the relationship between economic disadvantage and mental illness in high-income OECD countries. First, we synthesize official economic data on gender differences in relative poverty to investigate whether women experience higher exposure to relative poverty. Second, we conduct a systematic review and meta-analytic assessment of the association between income inequality and mental illness, examining gender as a potential moderator, to investigate their potentially greater vulnerability to the mental health consequences of economic inequality.

## 2 Methods

According to the aims of this article (see 1.4.), we conducted a two-step synthesis combining (a) descriptive analyses of official economic indicators and (b) a systematic review and meta-analysis of epidemiological studies. The review followed PRISMA guidelines (26) and the PRESS checklist for search strategy development (27). The review protocol was preregistered on PROSPERO (CRD420251123692).

### 2.1 Sex differences in relative poverty

We analyzed country-level data on relative poverty rates obtained from OECD harmonized statistics (28). The analytical sample covered the period from 2010 to 2025. Countries were included once they met the World Bank high-income classification (29), allowing for a staged inclusion of countries over time. This approach ensured that cross-national comparisons were restricted to economically comparable settings while maximizing temporal coverage. Countries that had not attained high-income status by 2010 were included from the first year in which high-income classification was reached. Observations from years prior to high-income status were excluded. The primary outcome was relative poverty, expressed as the percentage of individuals living below the national relative poverty threshold. All analyses were stratified by sex (female and male). Observations with missing values for sex or poverty indicators were excluded.

#### 2.1.1 Statistical analysis

Descriptive analyses were conducted using boxplots to visualize the distribution of relative poverty rates by sex across countries and over time.

Descriptive analyses were conducted using boxplots to visualize the distribution of relative poverty rates by sex across countries and years. Because female and male poverty rates were derived from the same country-year observations, sex-specific estimates were treated as paired observations rather than independent groups.

To estimate sex differences while accounting for the repeated measurement structure of the data, we fitted linear mixed-effects models with relative poverty rate as the outcome, sex as the primary fixed effect, and random intercepts for countries to account for within-country correlation. Year was included as a fixed effect to account for secular trends in poverty rates over time. Standard errors were estimated to account for the non-independence of observations within countries.

As a sensitivity analysis, we additionally conducted paired comparisons of female and male poverty rates within countries and years. To assess whether the COVID-19 pandemic influenced sex differences in relative poverty, analyses were repeated after stratifying the observation period into COVID (2020–2022) and post-COVID (≥2023) periods. Sex differences during these periods were evaluated using models accounting for the paired country-year structure.

### 2.2 Systematic review and meta-analysis

#### 2.2.1 Search strategy

We conducted a comprehensive literature search using electronic databases, including Medline via Web of Science, Cochrane, PubPsych, Embase Classic + Embase via Ovid, and APA PsycINFO via EBSCOhost, covering publications up to December 2025 from the past 20 years. The literature search followed predefined search terms. Boolean operators (e.g., AND, OR) and database-specific filters (e.g., English language, published after 2004) were applied to refine the search. Please refer to Supplement 1 for the detailed search strategy.

#### 2.2.2 Inclusion and exclusion criteria

We included peer-reviewed quantitative studies conducted in high-income countries that:

1. examined relative poverty or income inequality as primary exposures;
2. reported mental health outcomes assessed either through clinical diagnoses based on established diagnostic criteria (e.g., ICD or DSM) or validated mental health symptom measures (e.g., depressive, anxiety, psychotic, or substance-use symptoms);
3. provided gender-disaggregated estimates or sufficient data to derive gender-specific results; and
4. were published in English or German.

We excluded studies focusing exclusively on absolute poverty, those reporting only non-diagnostic psychological distress or quality-of-life outcomes, qualitative designs, case reports, reviews, protocols, editorials, and opinion pieces. Studies of children, adolescents, adults, and older adults were eligible.

#### 2.2.3 Study selection, screening, and quality assessment

Titles and abstracts were screened in two stages. Full texts of potentially eligible studies were independently assessed by two reviewers; disagreements were resolved by discussion or consultation with a third reviewer. Study quality was evaluated using the Newcastle–Ottawa Scale (30). Sensitivity analyses considered studies at high risk of bias.

#### 2.2.4 Data extraction and narrative summary

Data were extracted into a standardized spreadsheet, including study design, setting, sample characteristics, exposure definitions, outcome measures, statistical models, and gender-specific effect estimates. Narrative synthesis summarized patterns across studies, with attention to age groups, measurement approaches, and contextual factors, and was used to contextualize quantitative findings.

#### 2.2.5 Meta-analysis

For studies providing sufficient quantitative data, effect estimates were extracted as reported (e.g., odds ratios [ORs], risk ratios [RRs], incidence rate ratios [IRRs], regression coefficients, prevalence differences, or mean differences) and converted to a common effect metric. To enable synthesis across heterogeneous study designs and outcome measures, all effect sizes were transformed to log odds ratios (log[OR]), with corresponding standard errors calculated or derived from reported confidence intervals or p-values.

For studies reporting odds ratios, the natural logarithm of the reported OR was used directly:


log(OR) = ln(OR)

with the standard error calculated from the 95% confidence interval (CI) as:


SE(log[OR])=(ln(upper CI)−ln(lower CI))/(2 × 1.96)

Where risk ratios were reported, they were converted to odds ratios using the baseline risk (event probability) in the reference group when available:


$$\text{OR}=\text{RR}\times \left(1\;-{\text{ P}}_{0}\right)/\left(1-\text{RR }\times {\text{ P}}_{0}\right)$$

where P_0_ represents the outcome risk in the non-exposed/reference group. When baseline risks were not available, risk ratios were approximated as odds ratios under the assumption that outcomes were sufficiently uncommon, and these conversions were examined in sensitivity analyses.

Incidence rate ratios were converted to odds ratios using reported outcome prevalence or incidence information where available. When such information was unavailable, IRRs were treated as approximations of relative odds under the assumption that the underlying event rates were low and that this approximation would introduce minimal bias. Regression coefficients and mean differences were converted using established approaches based on the reported outcome distribution and variance estimates. Standardized mean differences were transformed into log odds ratios using the standard conversion:


log(OR)=SMD×π/√3

For studies reporting prevalence differences or other absolute measures, conversions were performed only when sufficient information was available to estimate the corresponding odds ratio. Studies without adequate information for reliable conversion were not included in the quantitative synthesis.

Because several studies reported multiple, non-independent outcomes (e.g., stratified by gender, age group, or mental health endpoint), we conducted a multivariate random-effects meta-analysis, allowing correlated effect sizes from the same study to be modelled jointly while preserving within-study dependencies. Random-effects models were applied to account for between-study heterogeneity. Statistical heterogeneity was assessed using Cochran’s Q and the I² statistic. Pre-specified subgroup analyses examined age group (youth vs. adults vs. elderly), type of mental condition, and gender. Gender moderation was further evaluated using subgroup meta-analysis and meta-regression where sex-disaggregated estimates were available. Given the limited number of studies reporting Gini-index-based measures, analyses examining the association between income inequality and mental health outcomes using Gini coefficients were considered exploratory. Moderator analyses involving Gini-index estimates were conducted to investigate potential patterns of effect modification by demographic and clinical characteristics; however, these analyses were interpreted cautiously due to the limited number of available effect sizes and the increased risk of unstable estimates when examining multiple moderators. Findings from these analyses were therefore considered hypothesis-generating rather than confirmatory. Publication bias was assessed using funnel plots and Egger’s regression test where applicable. All analyses were conducted in R (packages meta and metafor).

## 3 Results

### 3.1 Gender exposure differences in relative poverty

The finalized analytical sample consists of 102 country-year observations drawn from 24 high-income economies. The countries represented in this longitudinal dataset include Austria, Belgium, Czechia, Denmark, Estonia, Finland, France, Germany, Greece, Hungary, Iceland, Ireland, Italy, Latvia, Lithuania, Luxembourg, Netherlands, Norway, Poland, Slovakia, Slovenia, Spain, Sweden, and Türkiye.

**Figure 1:**
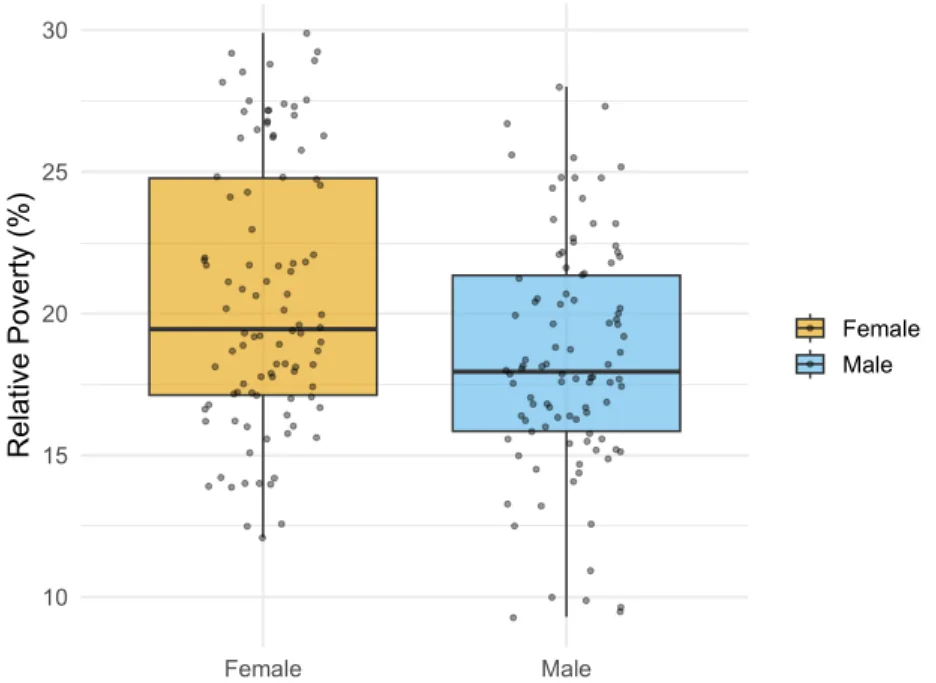
Box plot of gender differences in relative poverty across high-income OECD countries.

Across this high-income sample, relative poverty rates were consistently and significantly higher among women than among men. For the full observation period of 2020–2024, the mean relative poverty rate was 20.5% among women, compared to 18.3% among men. This represents a mean gender poverty gap of 2.18 percentage points (Welch t-test: t(197.9) = 3.56, p < 0.001). Please refer to **Figure 1** for the box plot.

Sensitivity analyses conducted on the post-pandemic period (> 2023, n = 35) confirm the persistence of this trend. In these most recent years, the mean gender gap remained nearly identical at 2.19 percentage points (p = 0.028). While individual observations in specific contexts (notably Sweden and the Slovak Republic) showed instances of gender parity or slight male-skewed poverty in single years, the aggregate data indicates that female sex remains a robust and consistent risk factor for relative poverty across the investigated settings.

### 3.2 Systematic review on gendered effects of economic inequality on mental conditions

**Figure 2:**
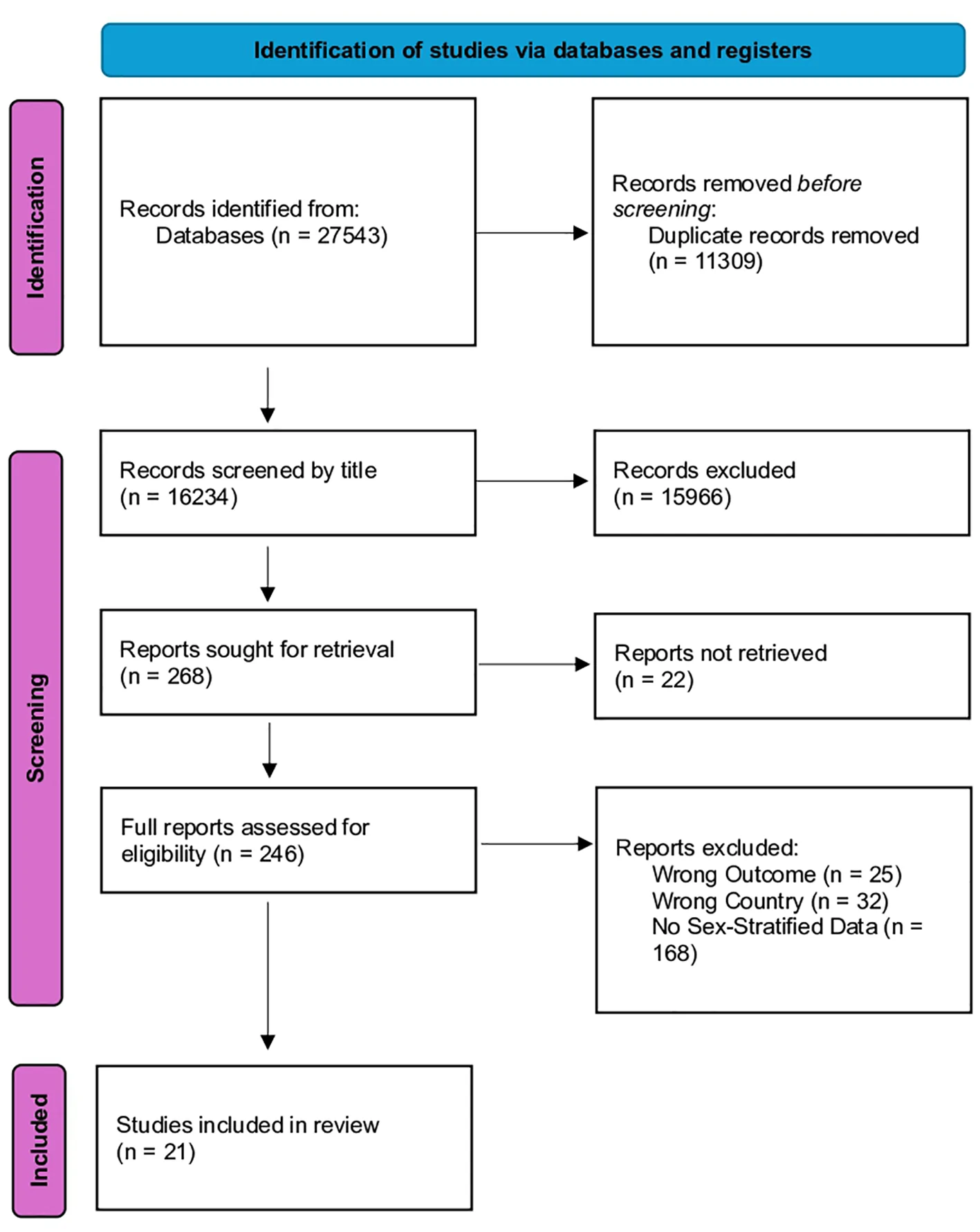
PRISMA flow chart of screened and included studies.

The literature search identified 27543 records through database (see **Figure 2**). After removal of 11309 duplicates, 16234 records remained for title and abstract screening. Of these, 15966 records were excluded. Finally, 21 studies met the inclusion criteria and were included in the qualitative synthesis.

#### 3.2.1 Study characteristics and geographic distribution

Across the 21 included studies (see **Table 1**), designs were predominantly cross-sectional (k=13), with five large longitudinal cohorts. Samples ranged from small clinical or community cohorts (n=685) to nationwide population registers exceeding four million individuals (n=4.396.518). The evidence base concentrated on the United States (k=9), Canada (k=3), Denmark (k=2), Japan (k=2), South Korea (k=2), Spain (k=1), UK (k=1), and multi-country European samples (k=1). Populations covered the life course, including adolescents, working-age adults, older adults, as well as specifically pregnant women and mothers in one study. Most studies operationalized economic position using individual household income (k=14), while a subset incorporated neighborhood or state-level Gini coefficients (k=8). Outcomes primarily focused on depressive symptoms or diagnosed depression (k=15), with several studies extending to anxiety (k=2), PTSD (k=1), alcohol dependence (k=1), psychotic disorders (k=2), and other outcomes (k=2). The methodological quality of the included studies was generally high, with Newcastle–Ottawa Scale scores ranging from 7 to 9.

**Table 1:** Summary of included studies on economic inequality and mental health.

Author (year)	Design	Setting/population	Sample size	Age	Outcome	Measurement	Income inequality measure	Adjusted variables	Effect for women	Effect for men
Andersen (2009)	Cross-sectional	Unemployed adults in Denmark	9254	36–54 years	Depression	Major Depression Inventory (MDI)	Household gross income	Sex, birth year, cohabitation, cohort and ethnicity	Minor depression prevalence: 8.1% Occupation grp 4–5: 1.6% MDD, 6.9% Minor Depression, 2.7% took Anti-Depressants unemployed: 10.4% MDD, 17.6% Minor Depression, 7.3% took Anti-Depressants	Minor depression prevalence: 6.5% Occupation grp 4–5: 2.0 MDD, 4,7% Minor Depression, 1.5% took Anti-Depressants unemployed: 12.4% MDD, 22.5% Minor Depression, 4.8% took Anti-Depressants
Benny (2022)	Intervention	Pregnant women in Canada	3387	18–47 years	Depression	Edinburgh Postnatal Depression Scale (EPDS)	Net household income	Household income and mother’s education level, age, ethnicity, ability to obtain prenatal care, sense of safety in her neighborhood, and sense of the level of crime in her neighborhood	34.4% were depressed at baseline	N.A.
Dennis (2021)	Cross-sectional	USA African American Adults	685	working-age adults	Depression; anxiety	PHQ-9; GAD-7	Household income	Age, sex, and caregiver status	For Unemployment: Depression: β = 0.32 (SE 0.1), p < 0.05 (Conditional effect of trauma: β =0.25, 95% CI: 0.08 - 0.33) Anxiety: β = 0.44 (SE 0.1), p < 0.01 (Conditional effect of trauma: β =0.30, 95% CI: 0.13 - 0.41) For Household Income: Depression: β = −0.13 (SE 0.1), p < 0.05 Anxiety: β = −0.12 (SE 0.1), p < 0.05	For Unemployment: Depression: β = -0.02 (SE 0.1), n.s. (Conditional effect of trauma: β =0.19, 95% CI: 0.04 - 0.25) Anxiety: β = 0.10 (SE 0.1), n.s. (Conditional effect of trauma: β =0.20, 95% CI: 0.06 - 0.31) For Household Income: Depression: β = −0.14 (SE 0.1), n.s. Anxiety: β = −0.07 (SE 0.1), n.s.
Gero (2017)	Cross-sectional	Japanese older adults	83100	>65	Self-reported depression	GDS-15	Relative deprivation; income rank (Yitzhaki index)	age, gender, years of education, marital status, number of diagnosed illnesses, self-rated health, and activities of daily living	Depression Rates (GDS≥5) in Q1 (lowest): 38.7% Q2: 32.9% Q3: 24.2% Q4 (highest): 19.3%	Depression Rates (GDS≥5) in Q1 (lowest): 41.8% Q2: 30.8% Q3: 25.2% Q4: 17.6%
Henderson (2004)	Cross-sectional	USA adults	42862	≥18 years	Depression; alcohol dependence	AUDADIS, DSM-IV Criteria	Gini coefficient	Gender, age, ethnic origin, education, income, family size, urbanity, beer tax	Alcohol Dependence (any symptom in past 12 months) Income <550: 13.5 (1.2) % Income 550–950: 9.9 (0.6) % Income 951–1300: 9.4 (0.6) % Income 1301–1700: 10.0 (0.7) % Income 1701–1999: 11.2 (1.0) % Income 2000–2999: 11.4 (0.5) % Income 3000–3999: 11.3 (0.7) % Income 4000–5999: 10.8 (0.8) % Income 6000–8999: 11.8 (1.1) % Income 9000+: 13.4 (1.2) % Depression (any symptom in past 12 months) Income <550: 32.4 (1.1) % Income 550–950: 32.4 (1.1) % Income 951–1300: 30.2 (0.9) % Income 1301–1700: 27.9 (1.0) % Income 1701–1999: 27.4 (1.5) % Income 2000–2999: 27.1 (0.9) % Income 3000–3999: 25.4 (1.0) % Income 4000–5999: 23.7 (1.1) % Income 6000–8999: 23.3 (1.5) % Income 9000+: 24.2 (1.7) % Any Depressive Symptom (past 12 months) by Adjusted Gini 0.295–0.327: 27.04 (1.17) % 0.328–0.337: 27.16 (1.18) % 0.338–0.347: 29.35 (1.29) % 0.348–0.360: 29.78 (0.99) % 0.361–0.374: 26.96 (0.72) % Any Alcohol Dependence Symptoms (past 12 months) by Adjusted Gini 0.295–0.327: 13.27 (1.21) % 0.328–0.337: 10.74 (0.59) % 0.338–0.347: 11.80 (0.90) % 0.348–0.360: 11.67 (0.79) % 0.361–0.374: 9.92 (0.40) %	Alcohol Dependence (any symptom in past 12 months) Income <550: 31.3 (2.3) % Income 550–950: 21.8 (1.2) % Income 951–1300: 22.5 (1.1) % Income 1301–1700: 19.5 (1.1) % Income 1701–1999: 21.7 (1.5) % Income 2000–2999: 21.4 (0.7) % Income 3000–3999: 22.0 (1.0) % Income 4000–5999: 21.2 (0.9) % Income 6000–8999: 19.9 (1.4) % Income 9000+: 24.0 (1.6) % Depression (any symptom in past 12 months) Income <550: 28.5 (1.6) % Income 550–950: 28.1 (1.5) % Income 951–1300: 27.1 (1.3) % Income 1301–1700: 26.6 (1.4) % Income 1701–1999: 25.2 (1.6) % Income 2000–2999: 22.8 (0.8) % Income 3000–3999: 20.8 (0.9) % Income 4000–5999: 19.8 (1.0) % Income 6000–8999: 20.3 (1.4) % Income 9000+: 18.8 (1.5) % Any Depressive Symptom (past 12 months) by Adjusted Gini 0.295–0.327: 20.26 (1.12) % 0.328–0.337: 26.32 (1.38) % 0.338–0.347: 22.73 (1.24) % 0.348–0.360: 25.62 (1.02) % 0.361–0.374: 22.52 (0.67) % Any Alcohol Dependence symptom (past 12 months) by Adjusted Gini 0.295–0.327: 23.78 (1.38) % 0.328–0.337: 21.86 (1.17) % 0.338–0.347: 22.00 (1.14) % 0.348–0.360: 23.55 (1.06) % 0.361–0.374: 21.07 (0.66) %
Hinata (2021)	Cross-sectional	Japanese adults	38499	40–74 years	Depressive symptoms	CES-D	Household income	sex, age, marital status, occupation, area, disease history, and ADL	Prevalence and odds ratios (ORs) for depressive symptoms according to income level (yen/year) Q1 (<3,000,000): 37.5%, OR: 1 (Reference) Q2 (3,000,000–5,999,999): 33.3%, OR: 0.84 (95% CI: 0.78–0.90) Q3 (6,000,000–8,999,999): 29.4% OR: 0.68 (95% CI: 0.62–0.75) Q4 (≥9,000,000): 28.4%, OR: 0.68 (95% CI: 0.60–0.77)	Prevalence and odds ratios (ORs) for depressive symptoms according to income level (yen/year) Q1 (<3,000,000): 34.6%, OR: 1 (Reference) Q2 (3,000,000–5,999,999): 29.2%, OR: 0.77 (95% CI: 0.71–0.83) Q3 (6,000,000–8,999,999): 26.1%, OR: 0.67 (95% CI: 0.60–0.75) Q4 (≥9,000,000): 22.4%, OR: 0.59 (95% CI: 0.51–0.68)
Jørring-Pallesen (2024)	Longitudinal	Danish population	4396518	≥30 years	Schizophrenia	ICD-10 diagnoses	Equivalized disposable income	Strong income gradient for schizophrenia	Unspecified dementia (Women) Q1 vs Q4: NV Q2 vs Q4: log_RR = 0.433 | CI 0.310–0.556 | Q = 0.00282 Q3 vs Q4: log_RR = 0.196 | CI −5.028–5.420 | Q = 0.00595 Delirium, not induced by alcohol and other psychoactive substances (Women) Q1 vs Q4: log_RR = 0.865 | CI 0.797–0.932 | Q = 0.00030 Q2 vs Q4: log_RR = 0.662 | CI 0.588–0.737 | Q = 0.00030 Q3 vs Q4: log_RR = 0.309 | CI 0.201–0.418 | Q = 0.00246 Other mental disorders due to brain damage and physical disease (Women) Q1 vs Q4: log_RR = 0.854 | CI 0.564–1.145 | Q = 0.00030 Q2 vs Q4: log_RR = 0.446 | CI 0.204–0.689 | Q = 0.00217 Q3 vs Q4: NV Mental and behavioral disorders due to sedatives or hypnotics (Women) Q1 vs Q4: log_RR = 1.657 | CI 0.537–2.778 | Q = 0.00554 Q2 vs Q4: log_RR = 1.686 | CI 0.230–3.141 | Q = 0.00481 Q3 vs Q4: NV Mental and behavioral disorders due to use of tobacco (Women) Q1 vs Q4: log_RR = 0.708 | CI 0.001–1.415 | Q = 0.00544 Q2 vs Q4: NV Q3 vs Q4: NV Persistent delusional disorders (Women) Q1 vs Q4: log_RR = 2.247 | CI 1.712–2.782 | Q = 0.00178 Q2 vs Q4: log_RR = 1.505 | CI 1.021–1.989 | Q = 0.00310 Q3 vs Q4: log_RR = 0.872 | CI 0.060–1.683 | Q = 0.00584 With regards to income in females, the strongest positive association was observed in schizophrenia (Q1 vs Q4, IRR 10·1 [6·1–17·2]) Bipolar affective disorder (Women) Q1 vs Q4: log_RR = 2.007 | CI 1.458–2.557 | Q = 0.00372 Q2 vs Q4: log_RR = 1.898 | CI 1.380–2.416 | Q = 0.00384 Q3 vs Q4: log_RR = 1.210 | CI 0.199–2.221 | Q = 0.00563 Depressive episode (Women) Q1 vs Q4: log_RR = 1.392 | CI 1.039–1.745 | Q = 0.00174 Q2 vs Q4: log_RR = 0.839 | CI 0.441–1.236 | Q = 0.00285 Q3 vs Q4: log_RR = 0.340 | CI −0.001–0.680 | Q = 0.00584 Recurrent depressive disorder (Women) Q1 vs Q4: log_RR = 1.399 | CI 1.112–1.686 | Q = 0.00032 Q2 vs Q4: log_RR = 0.989 | CI 0.673–1.304 | Q = 0.00188 Q3 vs Q4: log_RR = 0.359 | CI 0.066–0.651 | Q = 0.00500 Persistent mood [affective] disorders (Women) Q1 vs Q4: log_RR = 2.653 | CI 0.696–4.610 | Q = 0.00450 Q2 vs Q4: NV Q3 vs Q4: NV Other anxiety disorders (Women) Q1 vs Q4: log_RR = 1.002 | CI 0.787–1.218 | Q = 0.00178 Q2 vs Q4: log_RR = 0.554 | CI 0.308–0.800 | Q = 0.00244 Q3 vs Q4: log_RR = 0.307 | CI 0.061–0.554 | Q = 0.00572	Alzheimer’s disease Q1 vs Q4: log_RR = 0.448 | CI 0.355–0.541 | Q = 0.00224 Q2 vs Q4: log_RR = 0.454 | CI 0.378–0.530 | Q = 0.00180 Q3 vs Q4: log_RR = 0.381 | CI −8.564–9.326 | Q = 0.00236 Delirium, not induced by alcohol and other psychoactive substances Q1 vs Q4: log_RR = 0.992 | CI 0.886–1.098 | Q = 0.000286 Q2 vs Q4: log_RR = 0.613 | CI 0.550–0.677 | Q = 0.000288 Q3 vs Q4: log_RR = 0.267 | CI 0.144–0.390 | Q = 0.00387 Personality and behavioral disorders due to brain disease Q1 vs Q4: log_RR = 1.585 | CI 0.868–2.302 | Q = 0.00551 Q2 vs Q4: log_RR = 1.294 | CI 0.336–2.252 | Q = 0.00508 Q3 vs Q4: log_RR = 0.989 | CI −0.203–2.181 | Q = 0.00593 Mental and behavioral disorders due to use of tobacco Q1 vs Q4: log_RR = 0.753 | CI 0.123–1.383 | Q = 0.00456 Q2 vs Q4: NV Q3 vs Q4: NV Persistent delusional disorders Q1 vs Q4: log_RR = 2.878 | CI 2.240–3.515 | Q = 0.00294 Q2 vs Q4: log_RR = 1.694 | CI 1.055–2.333 | Q = 0.00488 Q3 vs Q4: log_RR = 1.564 | CI 0.087–3.041 | Q = 0.00464 Schizoaffective disorders Q1 vs Q4: NV Q2 vs Q4: NV Q3 vs Q4: NV Manic episode Q1 vs Q4: log_RR = 3.339 | CI 1.913–4.764 | Q = 0.00533 Q2 vs Q4: NV Q3 vs Q4: NV Bipolar affective disorder Q1 vs Q4: log_RR = 1.486 | CI 0.543–2.429 | Q = 0.00495 Q2 vs Q4: NV Q3 vs Q4: NV Depressive episode Q1 vs Q4: log_RR = 1.718 | CI 1.416–2.021 | Q = 0.000310 Q2 vs Q4: log_RR = 1.041 | CI 0.762–1.320 | Q = 0.00204 Q3 vs Q4: log_RR = 0.518 | CI 0.249–0.786 | Q = 0.00509 Recurrent depressive disorder Q1 vs Q4: log_RR = 1.699 | CI 1.373–2.025 | Q = 0.000313 Q2 vs Q4: log_RR = 1.040 | CI 0.752–1.328 | Q = 0.000315 Q3 vs Q4: log_RR = 0.613 | CI 0.312–0.915 | Q = 0.00397 Other anxiety disorders Q1 vs Q4: log_RR = 1.665 | CI 1.350–1.980 | Q = 0.00166 Q2 vs Q4: log_RR = 1.295 | CI 0.891–1.700 | Q = 0.00215 Q3 vs Q4: log_RR = 0.947 | CI 0.499–1.396 | Q = 0.00322 With regards to income, the strongest positive association was observed in schizophrenia (Q1 vs Q4, IRR 18–4 [8–5–39–9])
Lowe (2021)	Retrospective cohort	Mothers in Alberta	2461	>18	Anxiety; depression	Spielberger Anxiety; EPDS; CES-D	Gini coefficient	Ethnicity, Education, Marital status, Age, Neighborhood-level characteristics	Lower household income (≤ US$79,999 vs ≥ US$80,000) was consistently associated with higher anxiety symptoms across models. Effect sizes were stable and positive: β ≈ 0.20–0.22 95% CIs consistently excluded zero (approximately 0.10–0.31). The main effect of Gini was not statistically significant in any model: β ranged approximately from −0.03 to +0.02 All confidence intervals crossed zero. Time-varying effects (Months × Gini) were also non-significant. Lower household income was again consistently associated with higher depressive symptoms. Effect sizes were nearly identical to those for anxiety: β ≈ 0.20–0.22 95% CIs approximately 0.10–0.31, consistently excluding zero. Associations were stable across all models, including those adjusting for neighborhood characteristics. No significant association between Gini and depressive symptoms was observed: β estimates close to zero (approximately −0.01 to +0.01). Confidence intervals consistently crossed zero. Months × Gini interaction terms were also non-significant.	N.A.
Pabayo (2014)	Longitudinal	USA adults	34653	≥18 years	Depression	AUDADIS-IV, DSM IV	Gini coefficient	Age, sex, education, marital status, ethnic origin, income, urbanity, health perception, family history, life events	Gini Quintiles and Depression: Q1 is reference, Model 3-4 2nd Quintile: 1.13 (0.83,1.55); 1.18 (0.86,1.62) 3rd Quintile: 1.15 (0.87,1.53); 1.22 (0.91,1.62) 4th Quintile: 1.32 (1.00,1.76); 1.37 (1.03,1.82) 5th Quintile: 1.44 (1.10,1.88); 1.50	Gini Quintiles and Depression: Q1 is reference, Model 1-4 2nd Quintile: 1.03 (0.78,1.35); 1.12 (0.91,1.38); 1.04 (0.72,1.49); 1.02 (0.71,1.45) 3rd Quintile: 1.11 (0.90,1.37); 1.04 (0.85,1.28); 0.95 (0.68,1.34); 0.93 (0.66,1.31) 4th Quintile: 0.92 (0.76,1.10); 0.96 (0.76,1.21); 0.80 (0.53,1.22); 0.81 (0.54,1.23) 5th Quintile: 0.85 (0.73,0.99); 0.85 (0.62,1.17); 0.67 (0.41,1.10); 0.68 (0.42,1.12)
Rocha (2015)	Cross-sectional	Spain, adults	29476	>16 years	Mental Illness Symptoms	GHQ-12	Gini coefficient	Age, sex, social class, health coverage, origin, employment	Unemployed VS Working: 32.7% vs. 21.3% Unemployed OR 1.68 (1.44 - 1.95) Gini n.s. Results for women show that the increase in age, belonging to manual social class, being an immigrant from a low-income country and being unemployed/on sick leave or retired/pensioner were associated with a higher prevalence of poor mental health	Unemployed VS Working: 31.2% vs. 11.6% Unemployed OR 3.45 (2.47 - 4.82) Gini OR 137.8 (5.91 - 3210)
Zare (2022)	Cross-sectional	USA adults	11420	≥20 years	Depression	PHQ-9	Poverty-to-Income Ratio PIR	N.A.	Low-PIR: 1.54 *** [1.44–1.65] Medium-PIR: 1.28 *** [1.20–1.36] Covered by health insurance: 0.95 * [0.90–1.00]	Low-PIR: 1.19 *** [1.10–1.29] Medium-PIR: 1.01 [0.95–1.08] High-PIR: 0.77 *** [0.72–0.81] Covered by health insurance: 0.95 * [0.90–1.00]
Sánchez-Moreno (2021)	Cross-sectional	24 European Countries	68417	> 65 years	Depression	PHQ-9	Income quintiles, Gini coefficient	Sex, age, living alone, limitation in activities of daily living and general activity limitation	Lowest income quintile (Q1) with Depression: β = 0.30, 95% CI [0.14, 0.46], p <.001 Q2 with Depression: β = 0.15, 95% CI [0.01, 0.29], p <.05 Social Support with Depression: β = –0.12, 95% CI [–0.17, –0.06], p <.001 Income Q1 × Social support: β = –0.11, 95% CI [–0.18, –0.04], p <.001 Income inequality (Gini coefficient) and Depression: β = 8.56, 95% CI [–2.41, 19.53], n.s.	Lowest income quintile (Q1) with Depression: β = 0.43, 95% CI [0.27, 0.60], p <.001 Q2 with Depression: β 0.31, 95% CI [0.15, 0.46], p <.001 Social Support with Depression: β –0.20, 95% CI [–0.26, –0.13], p <.001 Income Q1 × Social support: –0.09, 95% CI [–0.17, –0.02], p <.01 Income inequality (Gini coefficient) and Depression: β = 12.97, 95% CI [1.61, 24.33], p <.05
Lee (2020)	Cross-sectional	Korea	10000	> 65 years	Depression	SGDS-K	Income quintiles	Age, living arrangement, residential area, employment status, number of chronic disease, physical function	2nd 25%: OR = 1.30 [1.01–1.67] 3rd 25%: OR = 1.75 [1.34–2.27] Lowest 25%: OR = 2.50 [1.92–3.24] Social participation contributed to buffer depressive symptom inequalities of 11.7% to 45.3% among women	2nd 25%: OR = 1.70 [1.12–2.57] 3rd 25%: OR = 2.02 [1.33–3.07] Lowest 25%: OR = 3.01 [1.99–4.57] Social participation contributed to buffer depressive symptom inequalities of 24.0% to 46.3% among men
Shim (2024)	Longitudinal	Korea	5176	> 60 years	Depression	CES-D	Household Income, Employment Status	Age, gender, marital status, religion, and education	Work main effect: −0.42*** (women) Work × social engagement: 0.12**	Work main effect: −0.54*** (men) Work × social engagement: 0.14**
Magee (2021)	Retrospective cohort	Individuals born 1990–1998 from Canada	193400	13–19	Psychotic disorders	Hospitalization or outpatient diagnosis (ICD-9/10)	Family and Neighborhood Income	Age, migration background, parent mental disorder	Low Family Income aHR: 1.97 (1.66, 2.35) neighborhood Quintiles (Q5 highest= Reference) Q4 0.81 (0.60, 1.08) Q3 1.07 (0.81, 1.40) Q2 1.0 (0.76, 1.32) Q1 1.26 (0.96, 1.64)	Low Family Income aHR: 1.70 (1.47, 1.96) neighborhood Quintiles (Q5 highest= Reference) Q4 1.18 (0.92, 1.50) Q3 1.17 (0.92, 1.50) Q2 1.42 (1.12, 1.79) Q1 1.41 (1.11, 1.79)
Pabayo (2016)	Cross-sectional	Adolescents from Boston	1878	12–17 years	Depressive symptoms	Modified Depression Scale (MDS)	Gini coefficient	Nativity, neighborhood income, social cohesion, crime and social disorder	Gini (Z-score) → Depression: OR 0.89 (0.76–1.06), Girls living in higher Gini-coefficient areas had significantly higher depressive scores than those living in more equal areas. An average depression score for girls from the most equal neighborhoods is 0.39 while the average score for girls in the most unequal neighborhoods is 0.50.	Gini (Z-score) → Depression: OR 0.79 (0.65–0.96) Income inequality was associated with a decreased likelihood of reporting high social cohesion among boys only and was not significantly associated with depressive symptoms.
Lee (2023)	Cross-sectional	Young adult men in the USA	1084	>18	Depression	CES-D	Total income	Age, marital status, and race/ethnicity	N.a.	Income: −0.36 (SD 0.09) 95% CI: −0.53, −0.18
Pabayo (2024)	Cross-sectional	Adolescents in grades 9–12 in Canada	74501	13–18 years	Online gambling	self-report	Gini coefficient		Gini (Z-score): 1.19 (0.95, 1.50), n.s. State median income (Z-score): 1.16 (1.06, 1.27)	Gini (Z-score): 1.12 (1.03, 1.22) State median income (Z-score): 1.11 (0.88, 1.41), n.s.

#### 3.2.2 Main findings (sex-stratified prevalence and gradients)

Lower individual income was associated with substantially higher prevalence or incidence of mental conditions in both women and men. Reported prevalence of depression ranged approximately from 28–39% in low-income women versus ~18–28% in high-income women, and from ~23–42% in low-income men versus ~18–25% in high-income men, depending on age group and setting (31–34). Among unemployed adults, Andersen et al. (35) observed major depression prevalences of 1.6% in women and 2% in men, with men showing higher rates within the same occupational group but women reported more antidepressant use (2.7% vs. 1.5%), hinting towards differences in healthcare access among men compared to women.

Several studies (36, 37) demonstrated stronger associations between low income and depression/anxiety in women, whereas others (31, 32, 38) found steeper gradients or larger effect sizes in men. Large registry data (39, 40) extended this pattern beyond affective disorders, showing pronounced economic gradients for severe mental illness: the lowest income quartile was associated with dramatically elevated risks of schizophrenia and other psychotic disorders (IRRs ≈10 in women and ≈18 in the total population for Q1 vs Q4), alongside graded increases for depressive and anxiety disorders. In contrast, contextual income inequality (Gini) showed mixed and generally weaker associations: some studies reported higher depression odds with greater inequality in women but not men (41), others found stronger effects in men (42), while several observed null independent Gini effects once individual income was accounted for (43).

#### 3.2.3 Differences across age categories

Age-specific patterns indicate that socioeconomic gradients operate throughout the life course but vary in expression. In adolescents and young adults, income inequality and low household income were associated with higher depressive symptoms or related behaviors, with notable sex interactions: girls showed higher depression scores in high-inequality areas (44), while low family income predicted increased risk of psychotic disorders in both sexes during adolescence (40). Among working-age adults, unemployment and low income were robustly linked to depression and anxiety, often with stronger emotional vulnerability observed in women (36), though some outcomes (e.g., unemployment effects in Spain) were more pronounced in men (42). In older adults (≥60–65 years), multiple studies from East Asia and Europe (31, 34, 38, 45) demonstrated strong, graded income effects on late-life depression, with odds ratios typically ranging from ~1.3 to >3.0 in the lowest income groups. Social participation, employment, and social support consistently buffered these associations in later life. Taken together, the evidence suggests persistent socioeconomic inequalities in mental health from adolescence to old age, with sex-specific sensitivities that change across populations.

### 3.3 Meta-analysis effects of income on mental conditions

#### 3.3.1 Overall association between income and mental health

**Figure 3:**
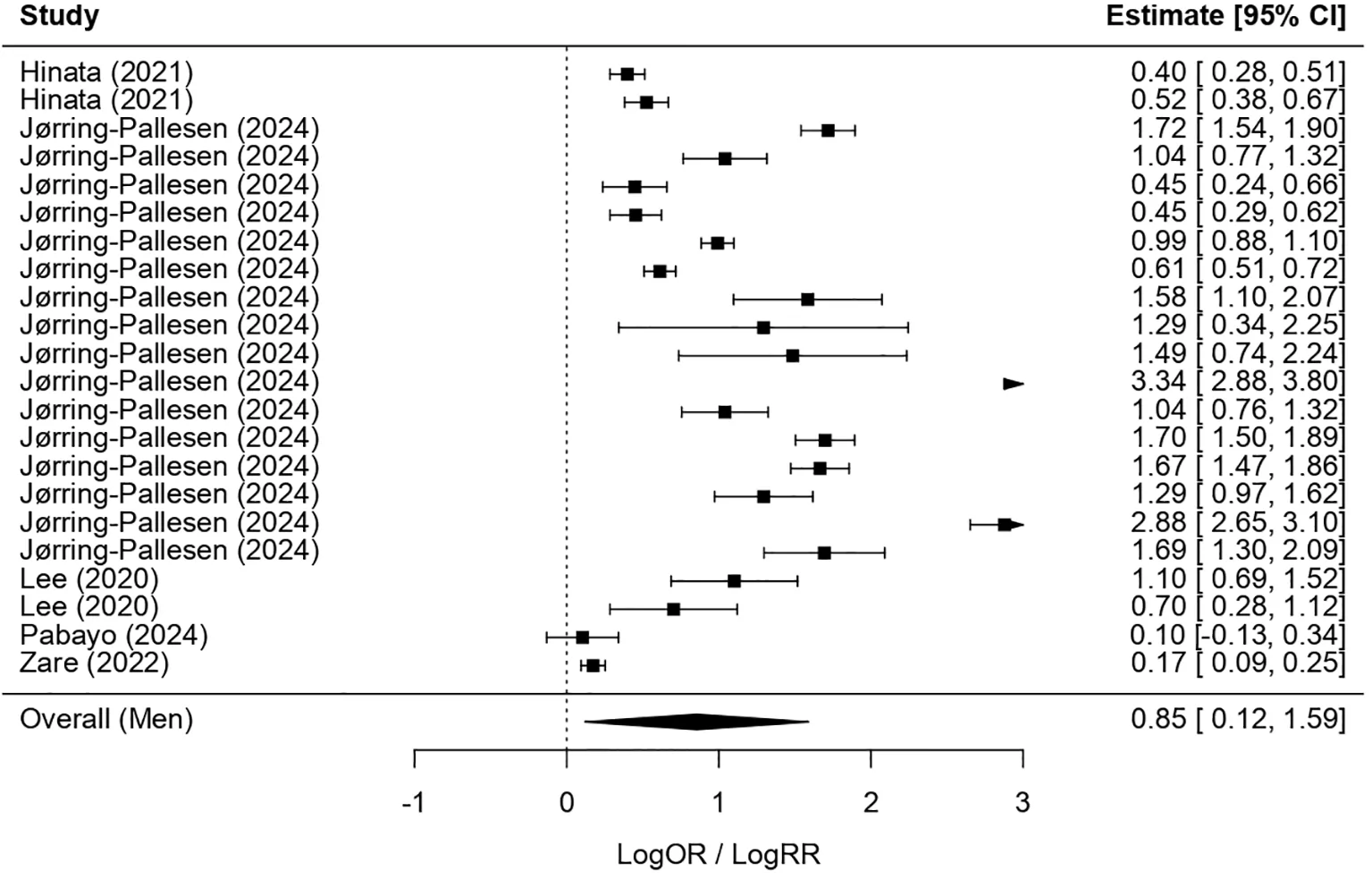
Forest plot including all outcomes among women.

**Figure 4:**
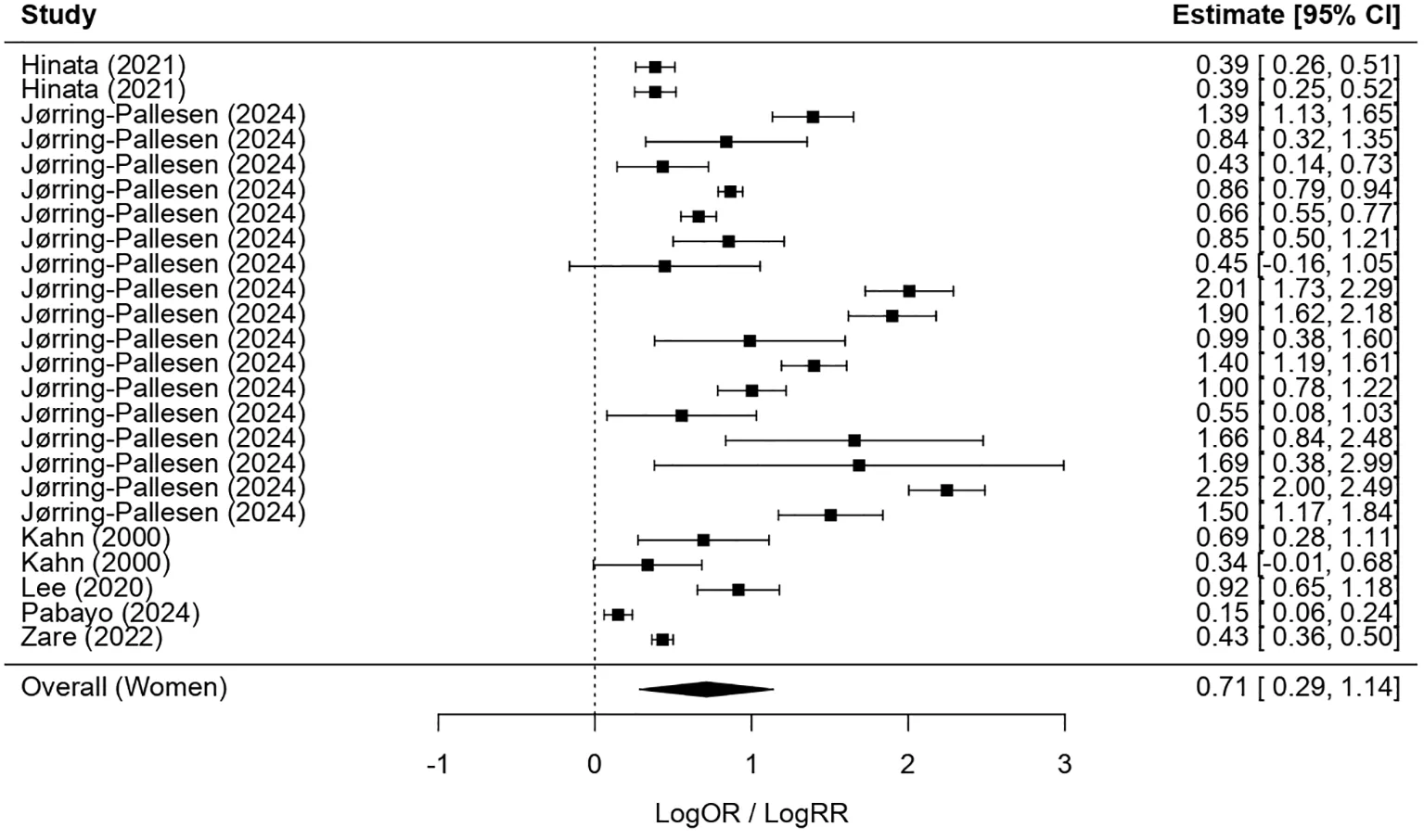
Forest plot including all outcomes among men.

Several studies reported multiple outcomes, hence 46 effect sizes were analyzed. Across 46 effect sizes, the multivariate REML model demonstrated a moderate and statistically significant pooled association between lower income and poorer mental health outcomes (estimate = 0.55, SE = 0.15, z = 3.65, p = 0.0003; 95% CI 0.26–0.85). Please refer to **Figures 3**, **4** for the gender-specific forest plots. Substantial between-study heterogeneity was observed (Q (45) = 1849.1, p < 0.0001), with variance attributable both to study-level clustering and outcome/gender nesting, justifying the multilevel specification. Gender was not a significant moderator in the overall model, including all mental conditions. Sensitivity analyses excluding studies with lower quality scores did not materially alter the pooled estimates, and no significant differences were observed according to study quality. Given the multivariate nature of the meta-analysis and the dependence among effect estimates, conventional assessments of publication bias (funnel plots and Egger’s regression tests) were not considered appropriate and were therefore not performed.

#### 3.3.2 Depression-specific analyses

Restricting the dataset to depression outcomes only (k = 17) yielded a pooled effect of 0.74 (95% CI 0.35–1.13, p = 0.0002). A gender moderator was statistically significant (p = 0.016), indicating smaller effects in men relative to women:

• Overall depression effect: 0.85 (95% CI 0.38–1.33, p = 0.0004)
• Gender moderator: −0.12 (p = 0.008)

Thus, women exhibited consistently larger income-related depression effects, supporting a sex-differentiated vulnerability pattern.

#### 3.3.3 Age and socioeconomic level as moderators

In the full dataset, neither age nor gender significantly moderated effects once all outcomes were included simultaneously, suggesting that sex differences are outcome-specific rather than global. Similarly, income level operationalization (individual vs state/neighborhood) did not significantly moderate pooled effects.

### 3.4 Meta-analysis effects of Gini index on mental conditions

#### 3.4.1 Overall association between income inequality and mental conditions

Across 9 effect sizes, the multivariate REML model showed no significant pooled association between income inequality (GINI) and mental health outcomes (estimate = −0.03, SE = 0.08, *z* = −0.35, *p* = 0.73; 95% CI −0.19 to 0.13). Despite the null pooled effect, significant between-study heterogeneity was observed (Q (8) = 28.47, *p* = 0.0004), indicating substantial variability across studies and outcomes.

#### 3.4.2 Gender as moderator

Including gender as a moderator significantly improved model fit (*QM* (1) = 4.36, *p* = 0.037). The gender coefficient was negative and statistically significant (β = −0.15, SE = 0.07), indicating smaller or inverse associations between GINI and mental health outcomes in men compared with women. Residual heterogeneity remained high (*QE* (7) = 28.42, *p* = 0.0002), indicating that gender alone did not account for observed variability.

#### 3.4.3 Fully adjusted model: gender, country, and income level

A final multivariate model including gender, country, and income level operationalization significantly explained heterogeneity (*QM* (3) = 11.27, *p* = 0.010).

In this model:

• The intercept (women, non-US samples, individual-level inequality) was positive and statistically significant (β = 0.27, *p* = 0.007), indicating a moderate association between income inequality and poorer mental health outcomes among women.
• Gender remained a significant moderator, with men showing significantly smaller effects (β = −0.17, *p* = 0.022).

Notably, residual heterogeneity in this fully adjusted model was no longer statistically significant (*QE* (5) = 4.60, *p* = 0.47).

## 4 Discussion

This study shows that gender shapes both exposure to economic disadvantage and vulnerability to its mental health consequences. Across high-income OECD countries, women experienced consistently higher relative poverty rates than men between 2010 and 2025, a gap that persisted before, during, and after the COVID-19 pandemic. Synthesizing 21 studies and 46 effect sizes, our multivariate meta-analysis further demonstrates a robust association between lower income and poorer mental health overall, with particularly strong effects for depressive outcomes. While gender did not moderate pooled effects across all psychiatric conditions, depression-specific models revealed significantly larger income-related effects among women, especially under maximal exposure contrasts (lowest vs highest income quartile), indicating sex-differentiated vulnerability in affective disorders. In contrast, income inequality measured via the Gini index showed no overall pooled association with mental health; however, gender emerged as a significant moderator, with inequality-related mental health burdens concentrated primarily among women.

Women’s disproportionate exposure to poverty has long been a central topic in social epidemiology and economics, with a large body of literature documenting gendered patterns of economic disadvantage (46, 47). However, findings regarding the magnitude and consistency of this gap remain mixed: while some studies report minimal or non-significant sex differences, others identify persistent female overrepresentation among low-income populations (48, 49). Therefore, our results must be understood within the temporal (2010–2025) and geographic restraints (high-income OECD countries) we have set in advance (due to the reasons explained in section 1.2.).

While previous research has not specifically examined the role of gender in the relationship between economic disadvantage and mental conditions overall, some studies have explored gender differences in specific disorders, such as depression. In the present study, we extend this literature by considering any mental condition as the primary outcome, while additionally conducting separate analyses focusing on depression. Yu (50), for instance, examined the association between the female-to-male ratio of depressive disorders and country-level indicators, including gross domestic product (GDP), the Gini index, and the Gender Inequality Index across 122 countries (50). The author reported that higher income inequality was significantly associated with depressive disorders among men, but not women (50). One reason for the findings, which contradict our own results, might be the lack of a geographical restriction in the analysis, as the study did not distinguish between effects in high-, middle-, and low-income countries. This might conceptually obscure the results, since in high-income countries, a higher Gini index typically reflects greater *relative* poverty, meaning that more individuals fall behind the societal median income while still meeting basic material needs. In contrast, in many low-income countries, a higher Gini index can coincide with widespread *absolute* deprivation, where large segments of the population lack essential resources, complicating interpretation.

Several included studies further suggest that employment status and work-related participation may exert stronger effects on mental health than income alone, pointing toward social rather than purely material mechanisms (35, 38, 42, 45). Unemployment and labor market exclusion were repeatedly associated with elevated depressive symptoms, implying that the psychosocial dimensions of work, such as daily structure, social integration, perceived purpose, and societal role expectations, may represent critical pathways linking socioeconomic disadvantage to mental illness.

We did not observe statistically significant moderation by age group in the pooled models, despite strong theoretical reasons to expect developmental differences. This null finding should not be interpreted as evidence of uniform mechanisms across the life course. Rather, it likely reflects limited power and heterogeneity within age-stratified subsamples. Importantly, children and adolescents experience socioeconomic disadvantage primarily through their parents, implying fundamentally different exposure pathways compared with adults. In these younger populations, mental health consequences are rather shaped by correlates such as, e.g. household stress, parental mental health, and educational environments, rather than individual income trajectories. Consequently, classic social drift explanations, whereby mental illness leads to downward socioeconomic mobility, are not applicable to minors, underscoring the predominance of social causation pathways early in life. These conceptual distinctions highlight the need for future research explicitly modelling age-specific mechanisms.

A similar logic applies to women living in households where men are the primary or sole income earners. In these contexts, women’s exposure to relative poverty and income inequality is often indirect, mediated through household resources rather than personal labor market participation. Consequently, mental health risks may arise less from individual income trajectories and more from relational and structural factors, including economic dependence, unequal bargaining power within households, caregiving burdens, and constrained social mobility. As with minors, this limits the applicability of social drift explanations and instead points toward social causation mechanisms operating through gendered divisions of labor and power. These dynamics further underscore the importance of distinguishing individual from household-level socioeconomic exposure when examining gendered mental health inequalities.

### 4.1 Limitations

Several limitations should be considered when interpreting these findings. First, our analyses are based on aggregate, country-level data for relative poverty and income inequality, which may obscure individual-level variation and limit causal inference. While meta-analytic pooling allows us to quantify general patterns, residual heterogeneity, particularly in the Gini analyses, indicates that unmeasured contextual factors may influence mental health outcomes. Second, study design heterogeneity among the included literature presents challenges: most studies were cross-sectional, which precludes strong conclusions about temporality, while longitudinal cohorts were limited in number and geographic scope. Due to the limited number of available studies for individual mental health outcomes, we were unable to perform separate meta-analyses for all diagnostic categories; therefore, we first assessed the broader outcome of any mental disorder and conducted an additional outcome-specific analysis for depression, for which sufficient evidence was available. The certainty of evidence for the association between lower income/income inequality and depression was assessed using the GRADE framework. Although the included studies consistently indicated an association between lower socioeconomic position and increased risk of depression, the overall certainty of evidence was rated as very low due to concerns regarding variability in exposure measures, study populations, analytical approaches, and outcome definitions. Third, although we focused on high-income OECD countries to reduce confounding by absolute poverty, this geographic restriction may limit the generalizability of findings to low- and middle-income settings, where the interplay between absolute and relative poverty could substantially differ. Fourth, sex-stratified and gender-sensitive analyses were inconsistently reported across studies, and definitions of poverty, income inequality, and mental health outcomes varied, introducing potential measurement bias. The frequent use of binary sex categories in the included studies limits conclusions about gender diversity. Finally, while our models explored moderators such as age, income level, and country, other relevant social determinants, including ethnicity, education, family composition, and social capital, could not be consistently included, leaving important sources of heterogeneity unexplained. The findings from the Gini-index analyses should be interpreted in light of several limitations. The number of available effect sizes was relatively small, limiting statistical power and increasing the potential for imprecise or unstable estimates, particularly when examining multiple moderators. Therefore, these analyses should be considered exploratory and hypothesis-generating rather than confirmatory. Future studies with larger samples and more consistent reporting of income inequality indicators are needed to further evaluate whether and under which conditions macro-level income inequality is associated with mental health outcomes. Despite these limitations, the study provides a robust examination of gendered patterns in the relationship between socioeconomic disadvantage and mental health within a well-defined, high-income context.

## Data Availability

The original contributions presented in the study are included in the article/**Supplementary Material**. Further inquiries can be directed to the corresponding author.
