# Supplementary material for: Gender differences in the effects of economic inequality on mental conditions: a multivariate meta-analysis of Gini index and relative income position in high-income OECD countries
Source: Front Psychiatry. 2026 Sep 3;17:1835495. doi: 10.3389/fpsyt.2026.1835495 (PMC13582524; doi:10.3389/fpsyt.2026.1835495)
Supplement: Supplementary file 1 [file Table1.docx]

Supplement 1:

Search Protocol

Medline via Web of Science

Search Terms

TS=(

(poverty OR "relative poverty" OR "income inequality" OR "low income" OR

"economic deprivation" OR "socioeconomic status" OR "social deprivation" OR

"income level" OR "income disparity")

AND

("mental health" OR "mental disorder*" OR "psychiatric disorder*" OR

depression OR anxiety OR "mood disorder*" OR "psychotic disorder*" OR

schizophrenia OR "bipolar disorder" OR suicide OR "suicide attempt*")

AND

(gender OR sex OR "sex difference*" OR "gender difference*" OR

male OR female OR men OR women OR "gender-specific")

)

Cochrane

Search Terms

(

(MeSH descriptor: [Poverty] explode all trees)

OR

(poverty OR "relative poverty" OR "income inequality" OR "low income" OR

"economic deprivation" OR "socioeconomic status" OR "social deprivation" OR

"income level" OR "income disparity"):ti,ab,kw

)

AND

(

(MeSH descriptor: [Mental Disorders] explode all trees)

OR

("mental health" OR "mental disorder*" OR "mental illness*" OR

"psychiatric disorder*" OR depress* OR anxi* OR

"mood disorder*" OR "psychotic disorder*" OR schizophrenia OR

"bipolar disorder" OR suicid* OR "psychological distress"):ti,ab,kw

)

AND

(

(MeSH descriptor: [Sex Factors] explode all trees)

OR

(gender OR sex OR "sex difference*" OR "gender difference*" OR

male OR female OR men OR women OR "gender-specific"):ti,ab,kw

)

PubPsych

Search Terms

(

poverty OR "relative poverty" OR "income inequality" OR "low income" OR

"economic deprivation" OR "socioeconomic status" OR "social deprivation" OR

"income level" OR "income disparity"

)

AND

(

"mental health" OR "mental disorder*" OR "mental illness*" OR

"psychiatric disorder*" OR depress* OR anxi* OR

"mood disorder*" OR "psychotic disorder*" OR schizophrenia OR

"bipolar disorder" OR suicid* OR "psychological distress"

)

AND

(

gender OR sex OR "sex difference*" OR "gender difference*" OR

male OR female OR men OR women OR "gender-specific"

)

AND

year:[2004 TO 2025]

Embase Classic + Embase via Ovid

Search Terms

(

'poverty'/exp OR

poverty:ti,ab,kw OR

"relative poverty":ti,ab,kw OR

"income inequality":ti,ab,kw OR

"low income":ti,ab,kw OR

"economic deprivation":ti,ab,kw OR

"socioeconomic status":ti,ab,kw OR

"social deprivation":ti,ab,kw OR

"income level":ti,ab,kw OR

"income disparity":ti,ab,kw

)

AND

(

'mental disease'/exp OR

"mental health":ti,ab,kw OR

"mental disorder*":ti,ab,kw OR

"mental illness*":ti,ab,kw OR

"psychiatric disorder*":ti,ab,kw OR

depress*:ti,ab,kw OR

anxi*:ti,ab,kw OR

"mood disorder*":ti,ab,kw OR

"psychotic disorder*":ti,ab,kw OR

schizophrenia:ti,ab,kw OR

"bipolar disorder":ti,ab,kw OR

suicid*:ti,ab,kw OR

"psychological distress":ti,ab,kw

)

AND

(

'sex factor'/exp OR

gender:ti,ab,kw OR

sex:ti,ab,kw OR

"sex difference*":ti,ab,kw OR

"gender difference*":ti,ab,kw OR

male:ti,ab,kw OR

female:ti,ab,kw OR

men:ti,ab,kw OR

women:ti,ab,kw OR

"gender-specific":ti,ab,kw

)

AND

[2004-2025]/py

APA PsycINFO via EBSCOhost

Search Terms

(

poverty OR "relative poverty" OR "income inequality" OR "low income" OR

"economic deprivation" OR "socioeconomic status" OR "social deprivation" OR

"income level" OR "income disparity"

)

AND

(

"mental health" OR "mental disorder*" OR "mental illness*" OR

"psychiatric disorder*" OR depress* OR anxi* OR

"mood disorder*" OR "psychotic disorder*" OR schizophrenia OR

"bipolar disorder" OR suicid* OR "psychological distress"

)

AND

(

gender OR sex OR "sex difference*" OR "gender difference*" OR

male OR female OR men OR women OR "gender-specific"

)

Search Date and Results:

| **Database** | **Host** | **Date of search** | **Number of search results** |
| --- | --- | --- | --- |
| MEDLINE | Web of Science | 13.12.25 | 16,211 |
| Embase Classic + Embase | Ovid | 13.12.25 | 1,280 |
| Cochrane Library | — | 13.12.25 | 0 |
| PubPsych | PubPsych.zpid.de | 13.12.25 | 1,004 |
| APA PsycInfo | EBSCOhost | 13.12.25 | 9,048 |
| **Total** |  |  | **27,543** |
| **After deduplication** |  |  | **16,234** |
| **After title and abstract screening** |  |  | **268** |
| **Full-text reports assessed for eligibility** |  |  | **246** |
| **Reports not retrieved** |  |  | **22** |
| **Reports excluded** |  |  |  |
| – Wrong outcome |  |  | 25 |
| – Wrong country |  |  | 32 |
| – No sex-stratified data |  |  | 168 |
| **Total full-text reports excluded** |  |  | **225** |
| **Studies included in the review** |  |  | **21** |
