# Supplementary material for: Gender differences in the effects of economic inequality on mental conditions: a multivariate meta-analysis of Gini index and relative income position in high-income OECD countries
Source: Front Psychiatry. 2026 Sep 3;17:1835495. doi: 10.3389/fpsyt.2026.1835495 (PMC13582524; doi:10.3389/fpsyt.2026.1835495)
Supplement: Supplementary file 2 [file Table2.docx]

Supplement 2: Summary of Study Quality (Newcastle-Ottawa Scale adjusted for cross-sectional studies)

| Author (year) | Representativeness of the cases | Sample size | Non-response rate | Ascertainment of the screening/surveillance tool | Potential confounders investigated by subgroup analysis or multivariable analysis | Assessment of outcome | Statistical test | Total |
| --- | --- | --- | --- | --- | --- | --- | --- | --- |
| Andersen (2009) | * | * |  | ** | * | ** | * | 8 |
| Benny (2022) | * | * |  | ** | * | * | * | 7 |
| Dennis (2021) | * | * |  | ** | * | * | * | 7 |
| Gero (2017) | * | * |  | ** | * | * | * | 7 |
| Henderson (2004) | * | * | * | ** | * | * | * | 8 |
| Hinata (2021) | * | * |  | ** | * | * | * | 7 |
| Jørring-Pallesen (2024) | * | * | * | ** | * | ** | * | 9 |
| Lowe (2021) | * | * |  | ** | * | * | * | 7 |
| Pabayo (2014) | * | * |  | ** | * | ** | * | 8 |
| Rocha (2015) | * | * | * | ** | * | * | * | 8 |
| Zare (2022) | * | * |  | ** | * | * | * | 7 |
| Sánchez-Moreno (2021) | * | * | * | ** | * | ** | * | 9 |
| Lee (2020) | * | * |  | ** | * | * | * | 7 |
| Shim (2024) | * | * |  | ** | * | * | * | 7 |
| Magee (2021) | * | * | * | ** | * | ** | * | 9 |
| Pabayo (2016) | * | * | * | * | ** | * | * | 8 |
| Lee (2023) | * | * |  | ** | * | * | * | 7 |
| Pabayo (2024) | * | * |  | ** | * | * | * | 7 |
