# Supplementary material for: Gender differences in the effects of economic inequality on mental conditions: a multivariate meta-analysis of Gini index and relative income position in high-income OECD countries
Source: Front Psychiatry. 2026 Sep 3;17:1835495. doi: 10.3389/fpsyt.2026.1835495 (PMC13582524; doi:10.3389/fpsyt.2026.1835495)
Supplement: Supplementary file 3 [file Table3.docx]

Supplement 3: GRADE assessment

| **Outcome** | **Studies/ participants** | **Design** | **Relative effect / association** | **Risk of bias** | **Inconsistency** | **Indirectness** | **Imprecision** | **Publication bias** | **Certainty of evidence** |
| --- | --- | --- | --- | --- | --- | --- | --- | --- | --- |
| Depression associated with lower income/income inequality | 12 studies  (>5 million participants) | Mostly cross-sectional; some cohort studies | Associations generally indicate higher depression risk with lower income (ORs approximately 1.2–3.0) | No serious risk-of-bias concerns identified; included studies were generally rated as moderate-to-high quality in the methodological appraisal | concerns (−1) due to variability in exposure measures (individual/household income, Gini index), populations, and effect measures | Some concerns (−1) due to heterogeneous populations and outcome definitions | No serious concerns (0) due to generally narrow CIs in large studies | Not formally assessed due to multivariate meta-analysis | **Very low** |
